# Investigation of the effect of PAn and PAn/ZnO photocatalysts on 100% degradation of Congo red under UV visible light irradiation and lightless environment

**DOI:** 10.3906/kim-1907-30

**Published:** 2020-04-01

**Authors:** Sibel ZOR, Bilge BUDAK

**Affiliations:** 1 Department of Chemistry, Kocaeli University Science-Art Faculty, Kocaeli Turkey

**Keywords:** Congo red, photocatalytic degradation, lightless, UV-vis light irradiation

## Abstract

PAn (polyaniline) and PAn/ZnO photocatalysts were synthesized using chemical polymerization of aniline. The structure characterization of the synthesized samples was analysed by using X-ray diffraction (XRD), Fourier transform infrared spectroscopy (FT-IR), scanning electron microscopy (SEM), transmission electron microscopy (TEM), and UV-Vis spectroscopy measurements, and thermal gravimetric analysis (TGA). The photocatalytic activities of PAn and PAn/ZnO (0.1g/100ml) on the degradation of the Congo red (CR) dye were studied under the UV visible light irradiation and the lightless environment and the efficiency of catalysts have been explained in details. Contribution of UV visible light irradiation on the 100% degradation of CR dye for the PAn and PAn/ZnO photocatalyst is determined. The reaction kinetics and effect of pH (pH 4 and pH 10) were investigated by using first order kinetic model. According to the experimental results, photocatalytic reaction rate of Congo Red increased in acidic environment and under UV visible light irradiation.

## 1. Introduction

Organic pollution is a big environmental problem and negatively effects eco-system and human health [1]. It is very necessary to remove organic pollutants from wastewater before being released into the natural environment. One of the most important environmental contaminants affecting the eco-environment, which are harmful to humans, plants, and animals in water, soil, and air are dyes. The presence of the dye in the receiving water avoids light leakage and reasons eutrophication, which disrupts the process of photosynthesis and prevents the growth of organisms in the water [2].

Congo red, dye selected as a candidate in this study, has a toxic effect, which causes severe health problems in humans and aquatic organisms affects all [3–7]. Benzidine is a metabolite of Congo red and causes cancer in the pancreas and human bladder. The effective and economical removal of this harmful dye from receiving water environments is very important. Adsorption of Congo red has been developed by using ozonation, biological treatment, various methods for the removal of the aqueous source containing sonochemical and photocatalytic decomposition in recent years. Among the available methods, photocatalytic application is more preferred because of its low cost, low temperature, and easy application [5–8].

Photocatalytic applications are an advanced oxidation processes to remove dye contaminants by a semiconductor photocatalyst and an energetic light source. The photocatalyst is a light induced activated catalyst. It absorbs light and becomes highly energized and transfers the energy to the reagents to initiate the chemical reaction [9, 10].

Inorganic nano metal oxide semiconductors (such as ZnO, TiO_2_ , CdS, and ZnS) have been used as the photocatalysts because of a large surface area, arrangement of electronic structure, light absorption ability, high-electron transfer characteristics and porous structures [11,12]. These semiconductors have a full band of valence and an empty conductivity band and act as sensitivity for light-induced redox processes. With the effect of irradiation, the electrons in the valence band pass through the conductivity band and form holes in the valence band. The electron-hole pairs may be recombined with each other or reacted one by one with other molecules. The holes may react with the electron donors in the solution or with the hydroxide ions to obtain highly oxidizing species such as hydroxyl or superoxide radicals [13–17].

Nowadays, nano catalysts may be used to improve the existing photocatalytic processes, i.e. to increase the photocatalytic effective sites. Polyaniline (PAn) as electroactive polymers have high conductivity, sufficient stability, simple synthesis procedure but also they are nontoxic and suitable composite component for semiconductors in their catalytic reaction [18–22]. Accordingly, nano modern science and photocatalytic processes as they form the polymeric composite of metal oxides with PAn in the development of specific applications are expected to play an important role.

PAn and metal oxide nanocomposites are promising as photocatalytic substances for the decomposition of various organic compounds [23]. In many photocatalytic environmental applications, the anatase form of TiO_2_ is used as semiconductor, the band gap energy in ZnO semiconductor is 3.37 eV, and it is shown as an alternative to TiO_2_ (band gap energy 3.2 eV) but also, ZnO nano metal oxides have unique catalytic, electrical, electronic, and optical properties. It is low-cost and offers a wide range of promising applications in the field of various nanotechnologies [21,22]. There are many reports on inorganic nanoparticles such as CdS [24] and CuS [25], clay and magnetite [26] for the preparation of PAn nanocomposites. Studies on photocatalytic degradation of different dye types on the surface of PAn/ZnO nanocomposite catalysts have been published. Using the PAn/ZnO nanocomposite for the photocatalytic degradation of the dyes, Saravan et al. found that, as a result of experimental studies, the degradation of the dyes was completed within 180 min [27]. In another study, MnO2 /PAn composites, methylene blue have been found to be effective in removing the photocatalytic methyl orange and rhodamine B [26]. Sharma et al. prepared PAn/ZnO nanocomposite film by a solution-casting method and investigated its dielectric properties [28]. They determined that the dielectric constant and loss factor of PAn were reduced in PAn/ZnO nanocomposite [29]. Ameen et al. used visible light radiation to study the photocatalytic activity of PAn/ZnO nanocomposite in the degradation of organic MB dye molecules. It was determined that photocatalytic degradation of MB increased [30].

In this study, Pure PAn and PAn/ZnO nanocomposites were prepared by chemical polymerization using ammonium persulfate (APS) as initiator. The structure characterization of the synthesized samples was done by FT-IR, XRD, SEM, TEM, and UV-Visible spectroscopy measurements. Thermal characterizations were performed with TGA. Accordingly, the effect of photocatalysts on 100% removal of dye was investigated in UVVisible light irradiation and lightless environments. Only the effect of UV-Visible light irradiation was calculated from the degradation results obtained. The photocatalytic degradation reaction kinetics was investigated in different pH environments to obtain the photocatalytic activity of the nanocomposites.

## 2. Materials and methods

### 2.1. Materials

Aniline (ANI, C6 H7 N) monomer had 99.5% purity, hydrochloric acid (HCl) 37%, ammonium persulfate (APS) ((NH4)_4_S_2_0_8_), ethanol (C_2_H_6_O) and sodium chloride (NaCl) were supplied by Merck and ZnO nanoparticles with an average particle size of less than 100 nm were purchased from Sigma Aldrich Company. Congo red (CR) dye (C_32_H_22_N_6_Na_2_O_6_S_2_) was from (CI 22120, BDH Chemical Ltd.) All reagents were of analytical grade and were used without further purification.

### 2.2. Synthesis of PAn and PAn/ZnO nanocomposites

PAn was obtained by chemical polymerization of aniline, APS was used as oxidant in the polymerization. In the polymerization of the aniline, 2 M HCl acid solution was prepared and 1 mL of aniline was added to 70 mL of 2 M HCl solution. The mixture was stirred in a magnetic stirrer at a constant speed for 2 h. The solution prepared by dissolving 2.5 g of APS in 100 mL of deionized water was added dropwise to the stirred solution at constant rate. Polymerization was continued at room temperature for about 5 h. The product was filtered by washing with 2M HCl solution, ethyl alcohol, and deionized water. The resulting solid product was dried at 60 °C, under vacuum for 4–5 h.

To prepare the PAn/ZnO nanocomposite, 0.1 g ZnO nanoparticles and 1 mL of aniline were added to a 70 mL of 2 M HCl solution. In order to prevent agglomeration of ZnO nanoparticles, it was stirred at constant speed for 1 h in the ultrasonic bath and 1 h in the magnetic stirrer, respectively. The solution prepared by dissolving 2.5 g of APS in 100 mL of deionized water was added dropwise to the stirred solution at constant rate. Polymerization was continued at room temperature for about 5 h. The product was filtered by washing with 2M HCl solution, ethyl alcohol, and deionized water. The resulting solid product was dried at 60°C, under vacuum for 4–5 h.

### 2.3. Photodegradation experiments

The photocatalytic degradation of Congo Red, PAn, and PAn/ZnO as catalysts were carried out under UV-A light (λ = 365 nm, UV-A 320 nm to 400 nm) irradiation. In the experiments, high pressure sodium lamp (OSRAM, VIALOX SON-T, 400 W) was used between 400 and 700 nm wavelengths. The maximum wavelength (λmax) for Congo Red (CR) was found to be 499–500 nm. Photocatalytic experiments were carried out at room temperature.

Photocatalytic degradation experiments of CR under UV visible light were made using UV cabinet at 365 nm wavelenght. Dye solution (50mg/L, 100 mg/L, and 150 mg/L) was prepared in deionize water and 0.1g of PAn and PAn/ZnO nanocomposite as photocatalysts were added into 100 mL of dye solution and the mixture was then stirred in lightless for 10 min to equilibrate. The UV light was turned on to start the photocatalytic degradation reaction. To ensure that the suspension was homogeneous, the solution was stirred for the duration of the experiment. After centrifugation of the degraded dye suspensions at 10.000 rpm for 5 min, the concentrations of the remaining dye solution were measured using UV spectrophotometer (Perkin Elmer Lambda 25). The photocatalytic degradation experiments of the dye were continued until 100% degradation occurred at specific time intervals. The same experiments were repeated in the lightless medium. According to Lambert Beer Law, calibration curve was obtained by plotting absorbance against the concentration of dye.

The degradation efficiency of the dye was calculated using the following equation [30, 31]:

(1)Degradation efficiency (%C)=((C0-C)C0)x100

C_0_, is the initial concentration of the dye and C, is the concentration of the dye after UV irradiation.

### 2.4. Characterization methods

Thermogravimetric analysis was performed under a dynamic nitrogen atmosphere (30 cm^3^ min^-1^) using micro and ultra-micro scale with a Mettler Toledo instrument at a heating value of 10 °C min^-1^ at a temperature range of 25–700 °C. FT-IR (Fourier transform infrared) spectra of compounds were obtained from the spectral Perkin Elmer system with a 2000 infrared spectrometer at 2000–600 cm^-1^ wavelength (in KBr Pellet). XRD (XRay diffraction method) measurements of the powder samples were made with a Bruker Advaced D8 XRD instrument. SEM (scanning electron microscope) analysis, the morphological and structural properties of the nanocomposites were characterized by using Cressington 208 C instrument and TEM (transition electron microscope) analysis JEOL-2100 LaB6 instrument. Photocatalytic degradation measurements were carried out by using Perkin Elmer UV-Lab Lambda 25 spectrophotometer.

## 3. Results and discussion

### 3.1. Characterization results of photocatalysts

**Figure 1:**
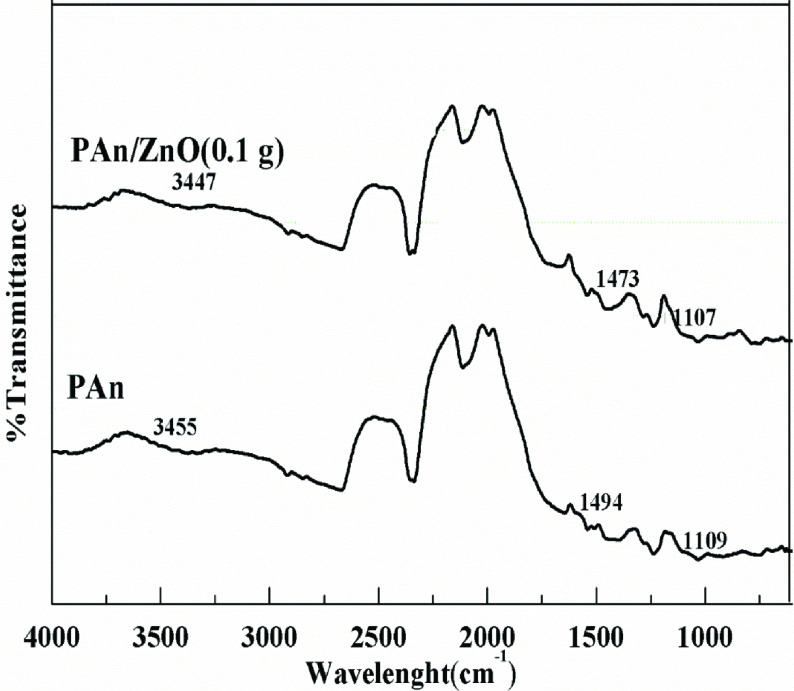
FT-IR spectra of PAn and PAn/ZnO polymeric nanocomposite.

FT-IR spectrum curves of chemically synthesized PAn and PAn/ZnO nanocomposite are shown in Figure 1. As shown in spectrum curves, the characteristic peaks of PAn can be attributed to: N-H stretching mode at 3455 cm^-1^ , C = N stretching mode of quinoid rings at 1583 cm^-1^ , C = C stretching mode of benzenoid rings at 1494 cm^-1^ , C-N stretching mode of benzenoid rings at 1291 cm^-1^ , plane bending vibration of C-H bond at 1109 cm^-1^ , the quinoid unit of PAn at 1041 cm^-1^ and C-C mode of aromatic rings at 797 cm^-1^ [32–34]. The peaks observed in the PAn/ZnO nanocomposite FTIR spectrum are the same with the PAn spectrum (Figure 1). However, by adding ZnO nanoparticles, the peaks corresponding to the PAn shifted to lower wavelengths [35–37]. Absorption bands of the PAn/ZnO polymeric composite are determined as the N-H stretching at 3401 cm^-1^ , C = N stretching mode of quinoid rings at 1577 cm^-1^ , C = C stretching mode of benzenoid rings at 1473 cm^-1^ , C-N stretching mode of benzenoid rings at 1290 cm^-1^ , C-H and C = C stretching mode of aromatic rings at 1108 cm^-1^ , the quinoid unit of PAn at 1039 cm^-1^ , plane bending vibration of C-H and C-C bonds at 791 cm^-1^ were determined [38,39]. These shifts in the peaks are due to differences in electron density and band energies between the PAn chain and the ZnO nanoparticles [38,40]. The presence of physicochemical interactions between PAn and ZnO molecules as well as hydrogen bonding between ZnO nanoparticles and PAn was determined by He. [41]. In addition, the shifts in the peaks in the FTIR spectrum indicate that ZnO nanoparticles enter the polymeric network of PAn.

**Figure 2:**
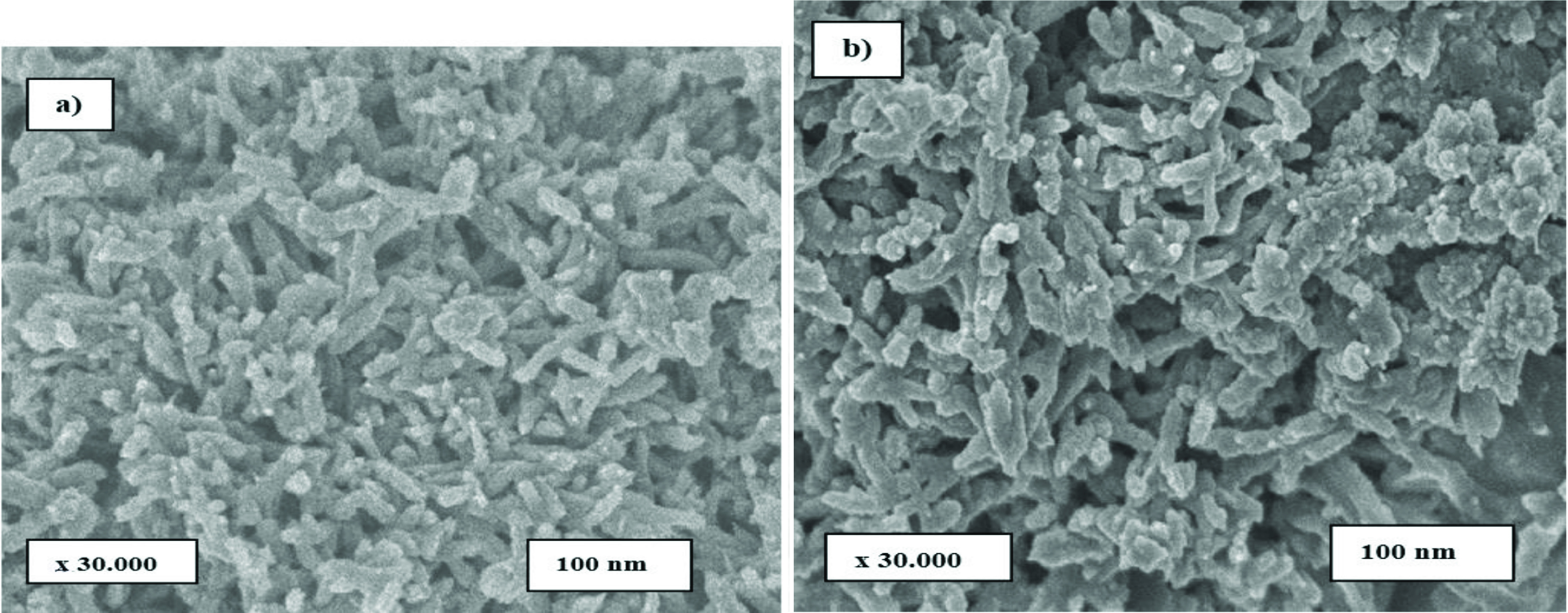
SEM photographs a) PAn and b) PAn/ZnO nanocomposite.

SEM photographs of the synthesized polymeric nanocomposites are given in Figure 2. According to the results, PAn nanocomposites have a spherical and homogeneous distribution (Figure 2a). Polymeric composites have added nano ZnO particles into the composite structure, causing clustering in some regions. The thin rod structure of the ZnO nanoparticles in the shape of a needle on the polymeric surface is also observed in the polymeric network structure (Figure 2b) [42]. The presence of a spherical structure is effective in increasing the adsorption capacity of the catalyst [43].

**Figure 3:**
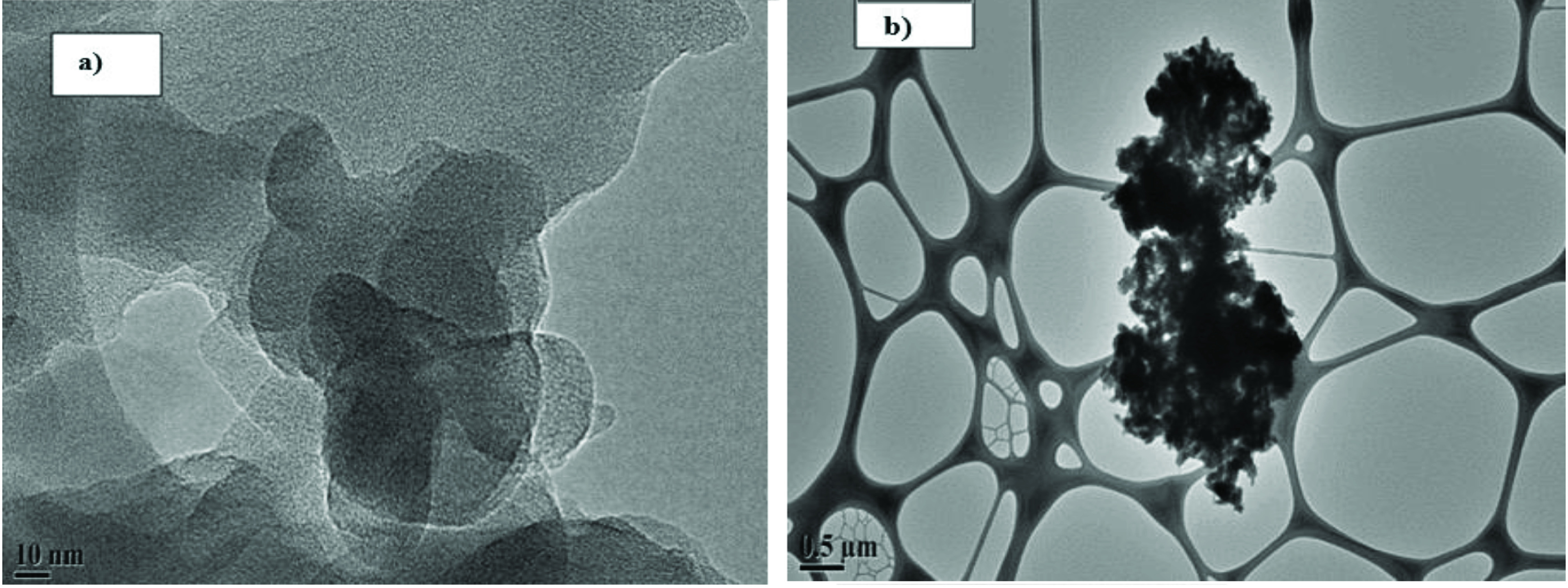
TEM images a) PAn and b) PAn/ZnO nanocomposite.

TEM images of the nanocomposites are presented in Figure 3. The characteristic network structure of PAn molecules is not doped with ZnO appeared in TEM results (Figure 3a). ZnO was incorporated into the polymeric network structure of PAn by addition of ZnO nanoparticles into PAn, and ZnO nanoparticles were involved in the thin rod-shaped polymeric network structure and agglomerate in some regions (Figure 3b).

**Figure 4:**
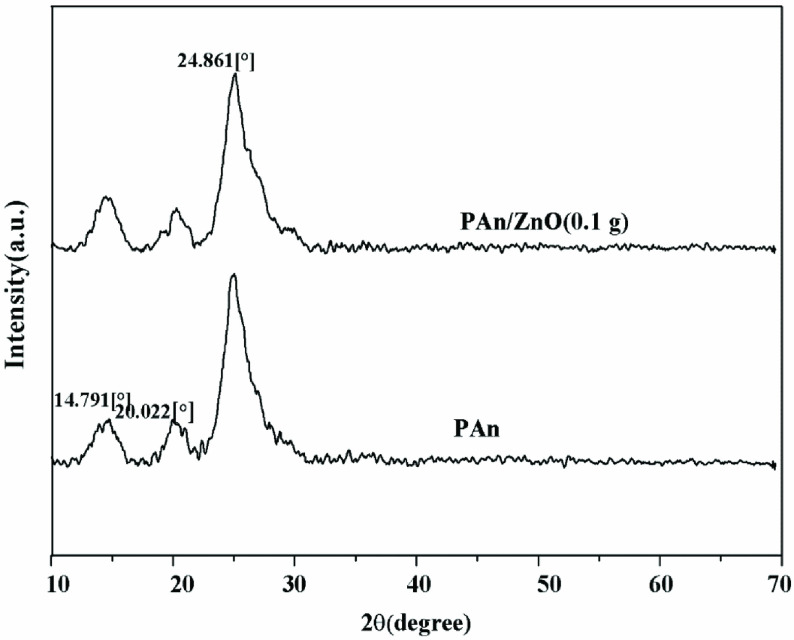
The XRD patterns of PAn and PAn/ZnO polymeric composites.

Figure 4 shows the XRD patterns of PAn and PAn/ZnO polymeric composites containing nano ZnO particles. For PAn powders, there are 4 characteristic peaks at 2θ angle. These are 14.8°, 20.02°, 24.86°, and 35.43°. For the PAn polymer, the 2θ degree is 20.3°and 25.1°in the literature, so the results obtained in this study are consistent with the literature. The distance among benzene ring planes or close contact between the adjacent chain links is showed by 2θ = 20°. In addition, the peak centred at 2θ = 25°may be detected to the scattering from PAn chains at interplanar spacing. It is generally expected that the polymers are in amorphous form, but the synthesized PAn polymers are crystalline due to the planar nature of the benzenoid and quinoid functional groups [44]. In literature studies, XRD pattern of ZnO nanoparticle exhibits the peaks at 2θ = 31.8°, 34.2°, 36.1°, 47.2°, 56.3°, 62.8°, 67.9°, 69.1°, and 70.2°are assigned to the (100), (002), (101), (102), (110), (103), (200), (112), and (201) lattice planes of ZnO hexagonal wurtzite phase [45,46]. In this study, XRD peak of ZnO nanoparticle did not appear because of less ZnO nanoparticle amount in polymeric matrix. In XRD models of PAn/ZnO nanoparticles, no significant shift was observed in PAn/ZnO nanocomposite prepared by adding 0.1 g of ZnO to the polymeric network structure. There are peaks indicating the presence of the ZnO nano-metal particle [44]. According to XRD results, ZnO nanoparticles were determined to be included in the polymeric network structure. Nanocomposite materials exhibit low crystallinity and small size due to interactions between PAn and ZnO nanoparticles [47–52].

**Figure 5:**
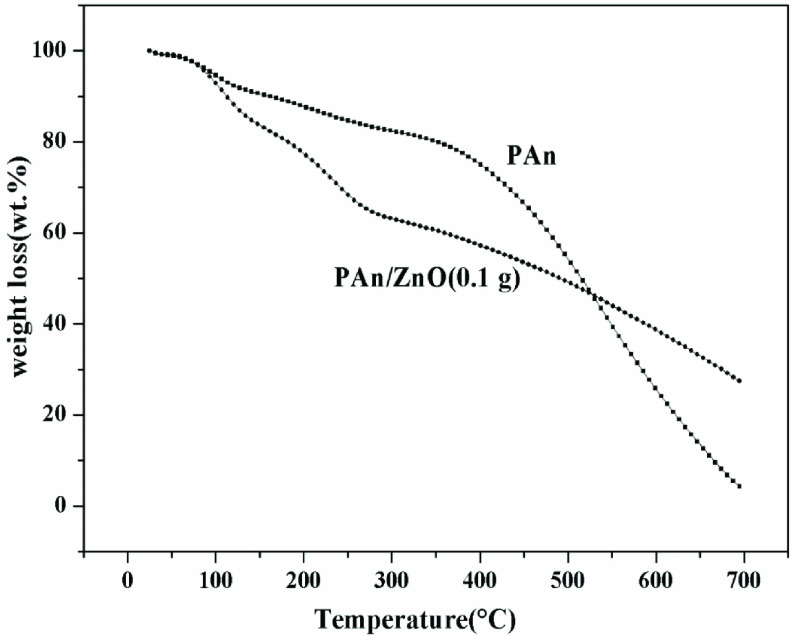
TGA curves of the PANI and PAn/ZnO nanocomposites.

The TGA results of PAn and PAn/ZnO nanocomposite are shown in Figure 5. As shown in Figure 5, PAn nanocomposites showed 16% of weight loss, due to the moisture and solvent absorbed at 235 °C. The nanocomposites containing ZnO had 3 stages of mass loss. The first stage was the removal of water, the second decrease was due to the release of anions, the third mass loss stage was the polymer originating from the dissociation [40]. Mass loss of PAn at 700 °C was 98%–99% and mass loss of PAn/ZnO was around 74%. The thermal equilibrium of the polymeric composite is higher than that of PAn. This is attributed to the presence of nano-ZnO particles in the polymeric matrix [53].

### 3.2. UV-Visible results

**Figure 6:**
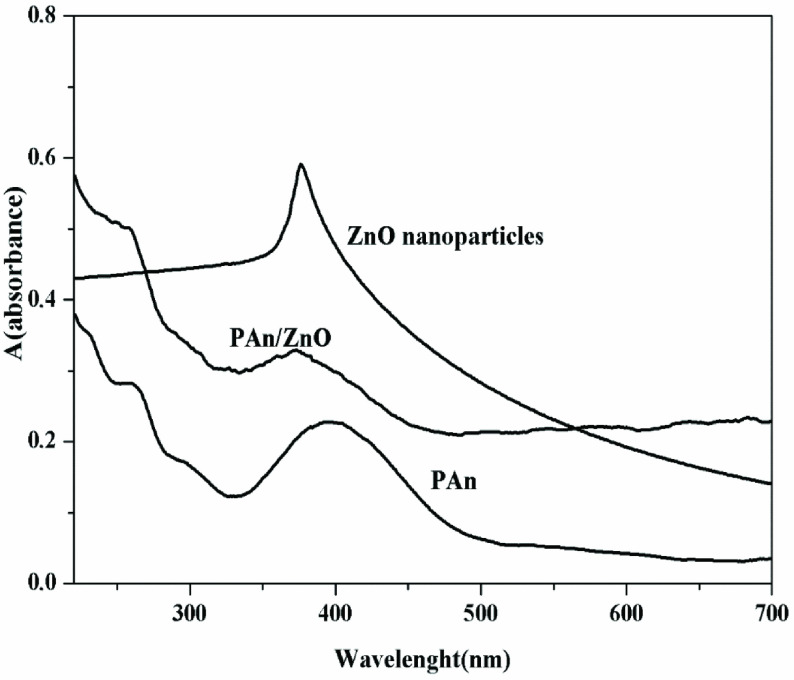
The UV-Vis spectrum curves of ZnO nanoparticles, PAn, and PAn/ZnO nanocomposite.

The UV-Vis spectrum of ZnO nanoparticles, PAn, and PAn/ZnO nanocomposites are shown in Figure 6. From the spectrum curves, ZnO nanoparticles absorb a sharp peak at a wavelength of 380 nm and strongly absorb UV light at this wavelength. That is, at this wavelength, electrons can be induced from the valence band of ZnO to the conductive band [54]. Previous studies showed that, PAn was determined to give UV spectrum bands in 3 regions. These are: π-π* transitions of band benzenoid groups at 320–360 nm, wide absorption bands at 400–440 nm, and 740–950 nm are concerned to doped level and polaron formation in conductive PAn (quinoid groups), respectively [50–56].

In this study, the broad band spacing of the Pure PAn form at 350-450 nm was associated with the π-π* transitions and polaron formation of the benzenoid groups. Accordingly, PAn can absorb both UV and visible light. PAn/ZnO composite shows the broad band range seen in PAn. ZnO can absorb both UV and visible light when covered with PAn, while absorbing only UV light alone. This case can be explained by the transfer of electrons from PAn to ZnO by means of hydrogen bonding between the PAn chain and ZnO molecules ((NH– (PAn) •••••O¦¦Zn) [54]. In other words, in PAn/ZnO photocatalyst under visible light irradiation, the electrons can be transferred to the conductivity band of ZnO nanoparticles from the LUMO band of PAn molecules [51, 54]. Accordingly, ZnO nanoparticles are photoactivated under UV light when used alone, while PAn/ZnO nanocomposite is photoactivated under harmless visible light irradiation [51].

In UV visible spectroscopy studies, the oxidation state of PAn in composites and the energy band gap of photocatalysts are important. According to the literature, pure ZnO, PAn, and PAn/ZnO’s band gap were determined as 3.2 eV, 2.82 eV, and 3.09 eV. Accordingly, the band gap value of the composite photocatalyst is less than that of pure ZnO. This indicates the interaction between PAn and ZnO, so the visible light absorbance of PAn/ZnO is correlated with the composite’s large absorption coefficient and small band gap. Because of the absorption of visible light of the solar spectrum, PAn/ZnO composite is recommended to be effective in degradation of pollutants in water [43,52].

### 3.3. Photocatalytic activity results

**Figure 7:**
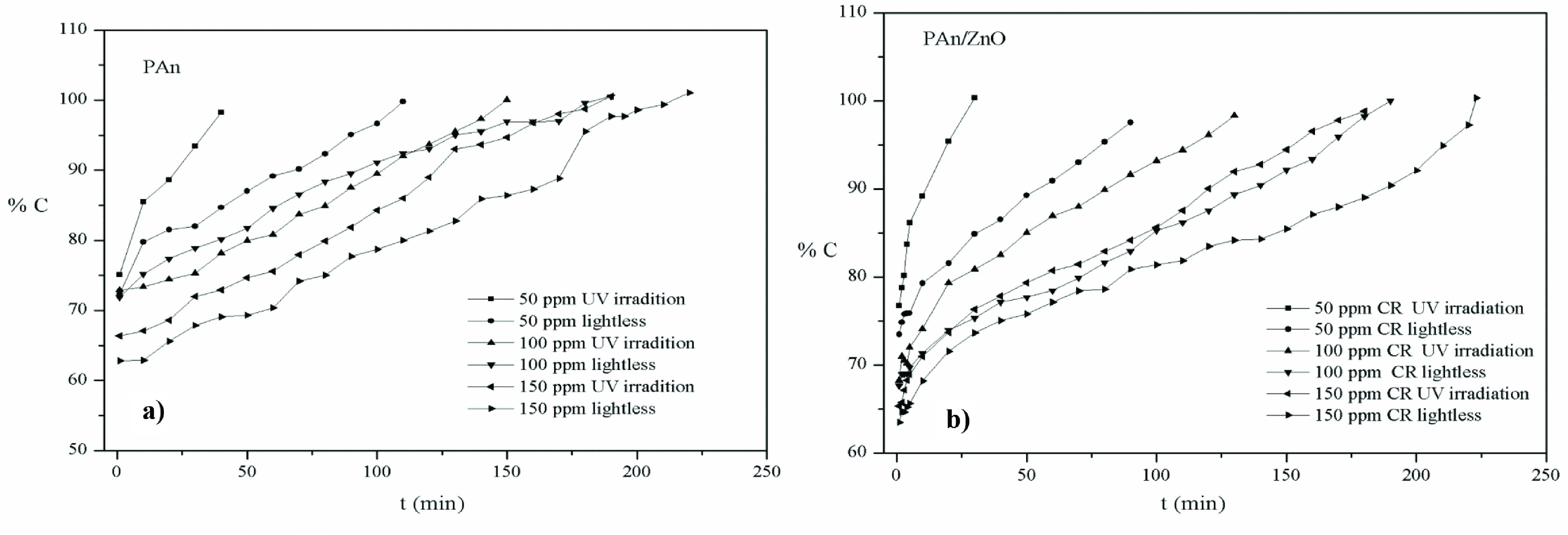
C% - t(min) graph in UV visible light irradiation and lightless medium for50ppm, 100ppm,and 150 ppm CR concentration for a)PAn, b) PAn/ZnO, photocatalysts.

Under the UV visible light irradiation and lightless conditions, photocatalytic activities of 0.1 g PAn and PAn/ZnO were investigated until 100% degradation in different concentrations of the CR dye solutions. The variation in degradation efficiency (C% ) of CR dye solutions with time intervals for PAn and PAn/ZnO nanocomposite under UV visible light irradiation and lightless is given in Figures 7a and 7b, respectively. Degradation efficiency of CR increases with time for both PAn and PAn/ZnO. The 100% degradation time of CR dye increases by the increase of the concentration of dye solution. Also, the 100% degradation time of CR decreases under UV-Visible light irradiation as compared to lightless environment. For PAn/ZnO catalyst, the degradation efficiency (C% ) of CR dye is higher than PAn (Figures 7a and 7b). In the degradation of the CR dye, the effect of only UV visible light irradiation is calculated from the following equation for photocatalyst, taking into account the degradation time.

The effect of UV vis. light irradiation = [(t, lightless) − t,UV light)] / (t, lightless)] x100 (2) t, _*lightless*_ is 100% degradation time of dye in lightless environment, t,_*UV light*_ is 100% degradation time of dye under UV visible light irradiation. UV visible light irradiation effect was calculated according to the obtained 100% degradation time results and presented in Table 1.

**Table 1:** 100% degradation time of CR dye and UV visible light irradiation effect.

	100% degradation time of CR, (under lightless) (min)	100% degradation time of CR, (under UV light irradiation) (min)	The effect of UV visible light irradiation (%) From Eq. 3
PAn			
50 ppm CR	115	40	65
100 ppm CR	190	160	16
150 ppm CR	225	195	13
PAn/ZnO			
50 ppm CR	90	30	67
100 ppm CR	180	130	28
150 ppm CR	215	180	16

The 100% degradation time of 50, 100, and 150 ppm CR was 115 min, 190 min, 225 min in the lightless environment in the PAn catalyst and 40 min, 160 min, 195 min under UV visible light irradiation, respectively. The effect of only UV light irradiation on the 100% degradation time of CR of PAn photocatalyst was determined as 65% for degradation of 50 ppm dye, 16% for degradation of 100 ppm dye, and 14% for degradation of 150 ppm dye (Table 1). The 100% degradation time of CR for PAn/ZnO photocatalyst in the lightless environment in 50 ppm, 100 ppm, and 150 ppm dye solutions is 90 min, 180 min, and 215 min, respectively, while under the UV-Visible light irradiation it is 30 min, 130 min, and 180 min, respectively. In the environment by using PAn/ZnO photocatalyst, UV-Visible light irradiation effect on 100% degradation of dye was determined as 67% in decomposition of 50 ppm dye, 28% in degradation of 100 ppm dye, and 16% in degradation of 150 ppm dye (From Eq. 2, Table 1).

PAn and PAn/ZnO photocatalysts decreased the 100% degradation time of dye under UV visible light irradiation. The photocatalytic activity of PAn and PAn/ZnO photocatalysts has been determined to be effective in 100% degradation of low dye concentration. As the concentration of dye is increased, the photocatalytic activity of the photocatalysts is decreased (Table 1). According to the results obtained, photocatalytic activity of PAn/ZnO is better than PAn both in UV visible light irradiation and lightless. This can be explained by increasing the active surface area of the PAn/ZnO composite by the addition of ZnO nanoparticles to PAn [52]. Also, while photocatalytic activity of the ZnO nanoparticles are effective only in UV light irradiation, when forms a compound with PAn, it appears that PAn is also effective in the UV-visible region by providing electron transfer to ZnO [51,57].

### 3.4. Photocatalytic reaction mechanism

**Figure 8:**
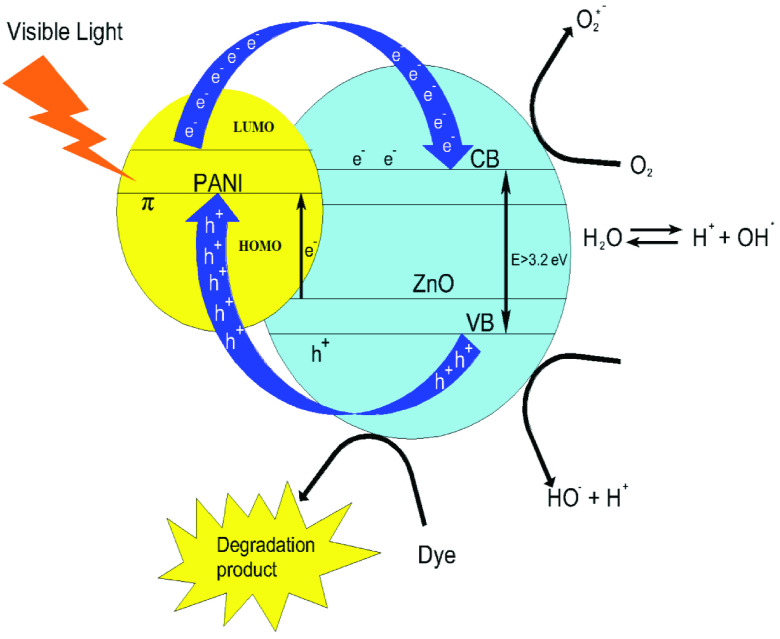
Schematic representation of the photocatalytic reaction mechanism of the PAn/ZnO photocatalyst.

The photocatalytic reaction mechanism of PAn/ZnO composite which is obtained by adding ZnO to PAn by considering the experimental results obtained in this study is presented in Figure 8 [58].

Accordingly, PAn molecules are stimulated by visible light irradiation and the electron is transferred to the conductivity band of ZnO nanoparticles (Eq. 3). The electrons in the ZnO’s conductivity band react with molecular oxygen to produce superoxide anion radical (Eq. 4). In the presence of these radical oxygen and organic molecules, hydrogen peroxide or organic peroxides form (Eq. 5, 6, and 7), hydrogen peroxide can be obtained in other ways (Eq. 8). Hydroxyl radicals, which are strong oxidants, can be formed by hydrogen peroxide (Eq. 9,10). The hydroxyl and oxygen radicals form the degradation products by interacting with the dye molecules (Eq. 11). According to this, ZnO nanoparticles are stimulated under UV irradiation to produce photocatalytic activity and electron hole pairs are formed [59, 60].

(3)PAn/ZnO + visible light →PAn•+/ZnO+eCB-

(4)eCB-+O2→O2•−

(5)H2O+O2•−→OOH•+OH-

(6)2OOH•→O2+H2O2

(7)O2•-+CR→CR-OO•

(8)OOH•+H2O+eCB-→H2O2+OH-

(9)H2O2+eCB-→OH•+OH-

(10)H2O2+O2•-→OH•+OH-+O2

(11)OH•/O2•-/PAn•++CR→degradation products

To ensure degradation of the dye molecules, the holes formed in the ZnO valence band can be carried to the PAn molecules to reach the photocatalyst surface in which they can achieve the dye molecules (Figure 8). The photogenerated electrons in the conductivity band of ZnO can react with oxygen to produce superoxide anion radicals that cause degradation of the dye. Therefore, PAn and ZnO increase the photocatalytic activity of each other with synergistic effect in combination [58]. In other words, PAn/ZnO composite under UV visible light irradiation has increased the photocatalytic activity for photodegradation of CR.

### 3.5. Kinetic results and the effect of pH

pH is one of the most important factors controlling the rate of reaction on the surface of the semiconductor. This parameter affects the degradation efficiency by changing the surface charge properties of the photocatalysts [59]. The photocatalytic degradation rate kinetics of the CR were investigated at the acidic and basic pH values (4 and 10) for the first 60 min using PAn and PAn/ZnO photocatalysts.

In general, the photocatalytic degradation of organic contaminants on semiconductor metal oxides is determined by using first order kinetic model [43,51].

(1)ln(CC0)=-kt

where k is the reaction rate constant, C0 is the initial concentration of dye, and C is the concentration of dye at time t.

**Figure 9:**
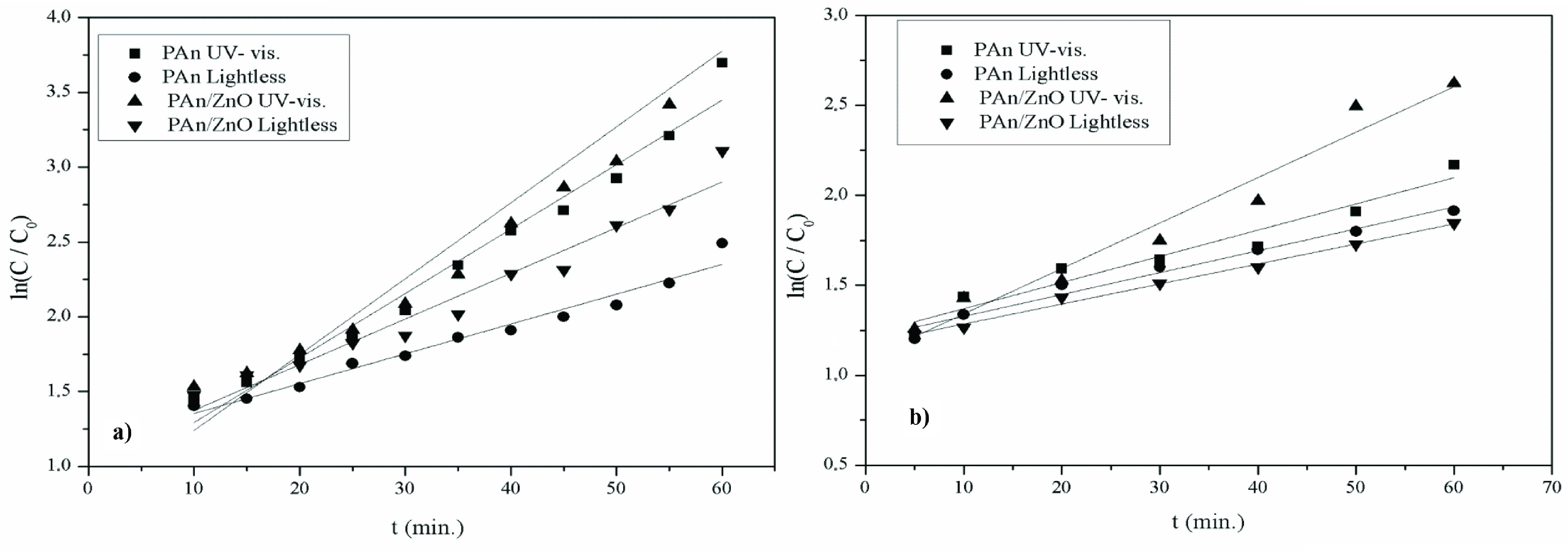
lnC /C0 -t graph for CR dye solution containing catalysis under UV visible light irradiation and lightless environment at different pH a)pH 4 b) pH 10.

In order to determine the photocatalytic reaction rate constant of CR under UV light irradiation and lightless environment, ln C/C0 versus time (t (min)) curves at different pHs (pH 10 and pH 4) are presented in Figures 9a and 9b. The reaction rate constants and correlation constants obtained by the linear correlation of ln (C/C0) -t curves are presented in Table 2. Accordingly, the k values in the UV visible light irradiation were higher than the k values in the lightless medium (Table 2).

In addition, the degradation reaction rate constants in PAn/ZnO photocatalyst were higher than PAn, and PAn/ZnO photocatalyst was found to be more effective in degrading CR dye. Photocatalytic degradation rate constants for PAn/ZnO increased under UV visible irradiation and at pH 4 as compared to PAn catalyst. The photocatalytic degradation reaction rate constants of CR decreased in basic environment and increased in acidic environment (Table 2). pH was found to be highly effective on degradation of dye molecules.

The pH of the photocatalyst varies depending on the net charge on the surface. Melaku Wondwossen et al. determined the point of zero charge (pzc) at the PAn/ZnO photocatalyst at pH 8.17 [61]. At the pH below the pzc, the photocatalyst surface has a net positive charge and above the pzc, net charge on it is negative [59,61]. The dye molecules in acidic solutions can easily be adsorbed onto the surface due to the electrostatic interaction between the negatively charged sulfo group molecule and the photocatalyst surface having a positive charge. Thus, photocatalytic degradation of the dye was increased in acidic medium (pH 4) by means of oxide and hydroxide radicals formed at low pH. At pH 10, due to the repulsion forces between the negatively charged sulfo group and the photocatalyst surface having a negative charge, the dye molecules cannot be easily adsorbed onto the photocatalyst surface. Therefore, the photocatalytic degradation of CR is reduced. Since the photocatalyst surface is negative in alkaline solutions and negatively charged in the dye molecules, adsorption of CR molecules to the PAn and PAn/ZnO catalyst surface is limited. Accordingly, the photocatalytic degradation efficiency of the dyestuff in basic media is lower than the acidic medium [54].

### 3.6. Stability test results

**Figure 10:**
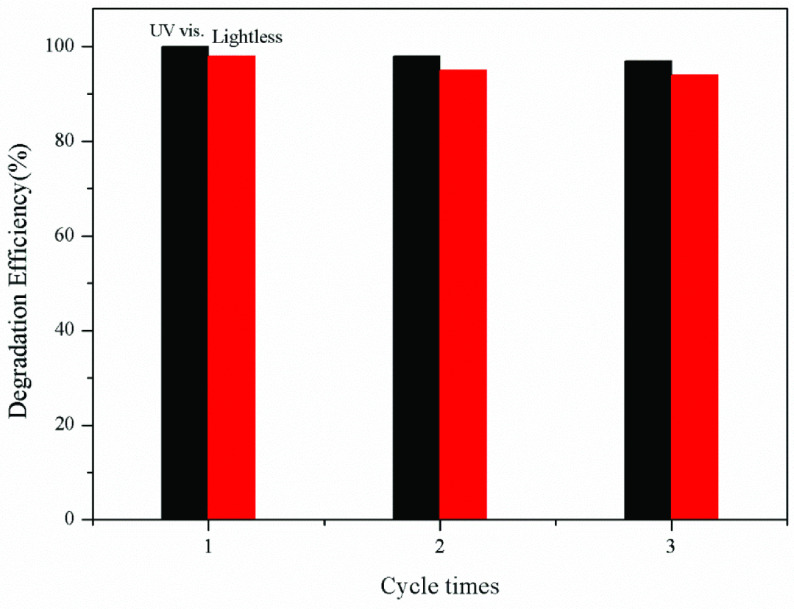
Reusability of the photocatalytic performance of PAn/ZnO under UV light irradiation and lightless.

The stability test of the synthesized PAn/ZnO photocatalyst was repeated 3 times for 100 ppm CR dye solution using the recovered photocatalysts. UV visible light irradiation and data obtained in lightless environment are given in Figure 10. For recycle study, after 3 cycles of photocatalytic process a slight decrease in the degradation efficiency is observed (Figure 10). This decrease can be explained by the partial inactivation of the surfaceactive sites of PAn and ZnO, or the partial destruction due to multiple washing [52]. This result indicates that PAn/ZnO have a good photocatalytic activity and easily recyclable, and reusable properties.

## 4. Conclusions

In this study, PAn polymer and PAn/ZnO nanocomposites are successfully synthesized and characterized by the chemical polymerization method. According to the results of the characterization, a strong interaction between PAn and ZnO nanoparticles has been determined. The most effective photocatalyst was determined as PAn/ZnO in both UV light and nonlight environment. The photocatalytic effect of the PAn/ZnO nanocomposite is associated to the synergistic effect between PAn and ZnO. The effect of UV visible light irradiation in degradation of 50 ppm CR dye is 65% for PAn photocatalyst and 13% for 150 ppm dye. The effect of UV light irradiation on degradation of 50 ppm CR for PAn/ZnO photocatalyst was 67%, while this ratio was 16% for 150 ppm dye. Therefore, UV-Visible light irradiation effect is decreased with increasing dye concentration. Photocatalytic degradation rate constants for PAn and PAn/ZnO under UV-Visible irradiation and at pH 4 were found as 0.0431min^-1^ and 0.051 min^-1^ . This indicates that the absorption capability of PAn/ZnO significantly increased due to the modification of ZnO nanoparticle with PAn. The PAn/ZnO catalyst is stable and can be used repeatedly. The simplicity and economy of the synthesis, the synergistic effect of the adsorption and photocatalytic effect, the degradation efficiency of the dye molecules increased. Therefore, these nanocomposites can be considered as a preferred alternative for waste water treatment.
